# Investigating anthelmintic efficacy against gastrointestinal nematodes in cattle by considering appropriate probability distributions for faecal egg count data

**DOI:** 10.1016/j.ijpddr.2017.01.002

**Published:** 2017-01-16

**Authors:** J.W. Love, L.A. Kelly, H.E. Lester, I. Nanjiani, M.A. Taylor, C. Robertson

**Affiliations:** a Department of Mathematics and Statistics, University of Strathclyde, 26 Richmond Street, Glasgow G1 1XH, UK; b Westpoint Research, Dawes Farm, Bognor Road, Warnham, West Sussex RH12 3SH, UK; c VParST Ltd, Wintringham. North Yorkshire YO17 8HX, UK; d Biomathematics and Risk Research, Animal and Plant Health Agency, New Haw, Addlestone, Surrey KT15 3NB, UK

**Keywords:** Cattle, Anthelmintic efficacy, Anthelmintic resistance, FECRT, Compound distributions, Zero inflated distributions

## Abstract

The Faecal Egg Count Reduction Test (FECRT) is the most widely used field-based method for estimating anthelmintic efficacy and as an indicator of the presence of anthelmintic resistant nematodes in cattle, despite never having been validated against the gold standard of controlled slaughter studies. The objectives of this study were to assess the normality of cattle faecal egg count (FEC) data and their transformed versions, since confidence intervals used to aid the interpretation of the FECRT, are derived from data assumed to be normally distributed, and violation of this assumption could potentially lead to the misclassification of anthelmintic efficacy. Further, probability distributions and associated parameters were evaluated to determine those most appropriate for representing cattle FEC data, which could be used to estimate percentage reductions and confidence limits. FEC data were analysed from 2175 cattle on 52 farms using a McMaster method at two different diagnostic sensitivities (30 and 15 eggs per gram (epg)) and a sensitive centrifugal flotation technique (SCFT) with a sensitivity of 1 epg. FEC data obtained from all egg count methods were found to be non-normal even upon transformation; therefore, it would be recommended that confidence or credible intervals be generated using either a Bootstrapping or Bayesian approach, respectively, since analyses using these frameworks do not necessarily require the assumption of normality. FEC data obtained using the SCFT method were best represented by distributions associated with the negative binomial and hence arithmetic means could be used in FECRT calculations.

Where FEC data were obtained with less sensitive counting techniques (i.e. McMaster 30 or 15 epg), zero-inflated distributions and their associated central tendency were the most appropriate and would be recommended to use, i.e. the arithmetic group mean divided by the proportion of non-zero counts present; otherwise apparent anthelmintic efficacy could be misrepresented.

## Highlights

• Efficacy can be misrepresented if inappropriate central tendencies are used in calculations.
• Sensitivity of counting technique used influences the distributions of best representation.
• Confidence intervals assuming normality of data would not be recommended to use with FEC data.

## Graphical abstract

**Figure:**
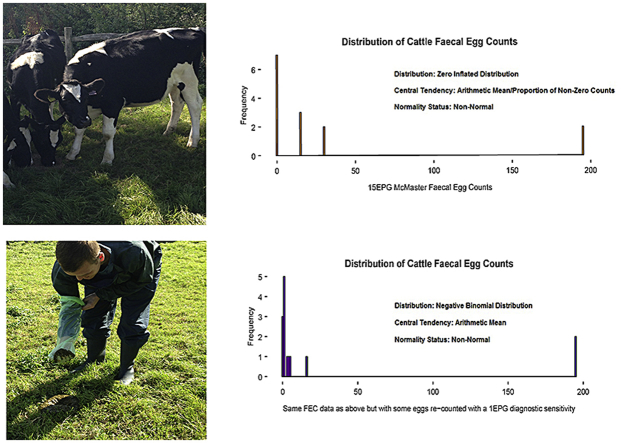

## 1 Introduction

For over 60 years the control of helminth parasites, due to their ever growing impact on animal health and welfare (Crofton, 1966, Vlassoff and McKenna, 1994, Corwin, 1997, Molento, 2009, Voort et al., 2013, Charlier et al., 2014), has increasingly relied on the use of anthelmintics. Many products are available worldwide and, for cattle, most are marketed for both treatment and prevention of helminthoses with the majority categorised into one of three broad-spectrum classes: benzimidazoles (1-BZ), imidazothiazoles (2-LV) and Macrocyclic Lactones (3-ML) (Taylor, 2010). Consequential to their continued use have been reports of apparent resistance to one or more of these classes of anthelmintics. Worldwide, the numbers of cattle herds thought to have been exposed to anthelmintic resistant helminths are not as alarming as the numbers for sheep flocks (Sangster, 1999, Kaplan, 2004, Wolstenholme et al., 2004, Waller, 1997) though resistance has been reported in Australia, New Zealand, parts of Europe and in some parts of the United States of America (Waghorn et al., 2006, Demeler et al., 2009, El-Abdellati et al., 2010, Edmonds et al., 2010, Sutherland and Leathwick, 2011). Although there have been no widespread reports of resistant helminths in cattle in the United Kingdom (UK), sporadic cases have been reported in the dose-limiting species, *C. oncophora* (Stafford and Coles, 1999, Sargison et al., 2009). Indeed, the true representation of resistance is difficult to assess mainly due to inconsistencies in treatment dose administrations, faecal sample collection and handling methods, faecal egg counting techniques used, associated experimental designs (Taylor, 2012) and the lack of robust methods for determining anthelmintic resistance under field conditions i.e. the lack of field data supported by controlled slaughter studies, or the availability of validated molecular and *in-vitro* methods for cattle nematodes.

Efficacy can be defined as a quantitative measure of the effectiveness of a drug intended to produce a desired effect (Vidyashankar et al., 2012). A fully effective anthelmintic is expected to reduce FECs to zero after administration of the anthelmintic. The most reliable method for determining anthelmintic efficacy is the controlled anthelmintic efficacy test, whereby animals are artificially infected, treated, then slaughtered and worm burden counts performed (Powers et al., 1982), but are not practicable in the field. It is common to assume that any apparent lack of efficacy is due to anthelmintic resistance – but this apparent resistance can be the result of anthelmintic failure due to other factors, most commonly under-dosing due to inaccurate estimation of bodyweight (Taylor et al., 2002). The most common method used to investigate anthelmintic resistance is the Faecal Egg Count Reduction Test (FECRT) (Coles et al., 1992, Coles et al., 2006). However, this test has not been validated against slaughter studies and the European Medicines Agency (EMA) regards this test as an estimation of efficacy, and not confirmation of resistance (EMA, 2014). True resistance must be confirmed through laboratory slaughter studies, potentially supported by molecular level studies, or methods such as egg hatch tests (Vidyashankar et al., 2012).

Faecal egg counts (FECs) provide an indirect measure of the worm burden present in cattle herds (and other livestock) since experimental studies have shown that there is a weak, positive correlation between FEC data and actual worm burden (Eysker and Ploeger, 2000). These counts, usually reported as the number of worm eggs per gram (epg) of faeces, can be obtained via a variety of methods. The McMaster technique and its modifications (Gordon and Whitlock, 1939, Whitlock, 1948, Ministry of Agriculture, 1986) are the most widely used and offer different egg detection limits, i.e. diagnostic sensitivities, typically ranging from 15 to 100 epg. For FEC methods with a high worm egg detection limit (low diagnostic sensitivity), a zero FEC may not necessarily correspond to no eggs being present; this is more likely to mean that the counting technique is not sufficiently sensitive to be able to detect any eggs present at or around the threshold of the egg detection limit. This is likely to result in false/excess zeros being present in FEC data and these can reduce the value of the arithmetic mean, i.e. the central tendency of the negative binomial distribution (Shaw and Dobson, 1995, Morgan et al., 2005, Denwood et al., 2008, Levecke et al., 2012), which is currently recommended for use in calculating percentage reductions when conducting a FECRT.

Areas of interest that exist, with regards to the statistical aspects of the FECRT, include the use and identification of appropriate experimental study designs (Lyndal-Murphy et al., 2014) and the analysis of FEC data (Presidente, 1985, Dobson et al., 2009). The objective of this study is concerned with the latter, since the purpose of this study was to determine whether or not current guidelines on parameter estimates and confidence intervals for estimating apparent anthelmintic efficacy are appropriate, using FEC data collected through an extensive field study. Firstly, the asymptotic assumption of normality of data, on which the confidence intervals are based, was assessed using these data. Secondly, various discrete probability distributions, such as *compound* distributions other than the negative binomial, were fitted to the data to determine the most appropriate distributions for representation. Based on the results, recommendations of possible alternative calculations are given.

## 2 Materials and methods

### 2.1 Field studies

All data used were collected between 1st September 2011 to 28 February 2015, i.e. over three full grazing seasons, from both dairy and beef farms throughout England. Farms were selected on the basis that they had adequate handling facilities and had not treated their first year grazing cattle with an anthelmintic prior to turn-out to pasture.

#### 2.1.1 Study design

Composite group faecal samples were collected approximately every two weeks from cattle on farms until the group mean FEC reached >150 epg. Once groups had reached this threshold, they were enrolled into a FECRT study. This threshold was chosen as it was unlikely to be high enough to cause clinical disease in individual animals, but still high enough for a robust FECRT assessment (Coles et al., 1992, Coles et al., 2006). These FEC screenings were carried out with ten cattle being sampled per forty cattle on a farm, where possible, and approximately 50 grams of faeces were retrieved from each individual animal. Composite samples from ten animals, each containing 3 grams of faeces from each were then examined using the Modified McMaster technique with a diagnostic sensitivity of 15 epg (MAFF, 1986).

For the FECRT, cattle at the start of the study (Day 0) were systematically allocated to either treatment or control groups as they came through the cattle crush. Fresh faecal samples were collected from all animals, placed into zip-lock bags, labelled with the individual ear tag numbers and refrigerated. Cattle in the treatment groups were dosed based on the individual body weights (kg), measured using either weightape or by electronic weigh scales, where available, using dose rates based on 10 kg increments (3-ML) or 13 kg increments (1-BZ). All cattle were returned to the same pastures so that they were subject to the same parasite challenge. Further faecal samples were collected 14 days post-treatment (Day 14). Control animals, which were not treated on Day 0, were treated after obtaining faecal samples on Day 14. Blinding of the laboratory technicians was maintained during faecal egg counting. On-farm treatments were administered using products either from the 1-BZ or 3-ML class of anthelmintics. The choice of anthlelmintic used was based on farm history and previous anthelmintic use. From the BZ group, an oral drench product containing fenbendazole (Panacur 10% Oral Solution™, MSD Animal Health, 7.5 mg fenbendazole/kg bodyweight) was used on 12 groups of cattle; and from the ML group, doramectin injection (Dectomax Injection for Cattle and Sheep, Elanco Animal Health Ltd, 200 mcg doramectin/kg bodyweight) was used on 19 groups of cattle, doramectin pour-on (Dectomax Pour-On for Cattle, Elanco Animal Health, Ltd, 500mcg/kg bodyweight) was used on 8 groups of cattle, ivermectin injection (Ivomec Classic Injection for Cattle and Sheep, Merial Animal Health, Ltd., 200mcg/kg bodyweight) and ivermectin pour on (Ivomec Classic Pour-On for Cattle, 500mcg/kg bodyweight) were also used on 15 and 7 groups of cattle, respectively. A positive or negative control group was used on all pastures, excluding those where pour-on products were used due to the likelihood of cross-contamination of controls with pour-on products. In total, 15 negative control groups were used. Treatment groups varied in size on farms throughout the study, with some farms having more than one positive treatment group enrolled into the FECRT. Based on the 61 positive treatment groups present in this study, the median group size for positive treatment groups was 27 cattle, with group sizes ranging between 12 and 58 cattle. For the 15 negative control groups present in this study, the median group size was 18 cattle, with group sizes ranging between 12 and 54 cattle.

### 2.2 Laboratory methods for obtaining faecal egg counts

Individual faecal egg counts were performed using a Modified McMaster counting technique (MAFF, 1986). Using 3 grams of faeces in 42 ml of water (1/15 dilution) and counting all eggs present in one or both chambers (0.5 or 1 ml), giving diagnostic sensitivities of 30 or 15 epg, respectively. However, any samples with a FEC< 120 epg (in 2012) or 60 epg (in 2013/14), were subsequently analysed using a *Sensitive Centrifugal Flotation Technique* (SCFT) method with a sensitivity of 1 epg (MAFF, 1986).

### 2.3 Farm data

We use the term *data* to describe each of one of the variations of the sets of egg counts that were obtained from the first and second McMaster chambers, using a diagnostic sensitivity of 30 epg (hereby referred to as 30EPG_McM1, 30EPG_McM2 data, respectively). The average of the two chambers was also considered, resulting in data sets with egg counts being obtained using a diagnostic sensitivity of 15 epg (hereby referred to as 15EPG_McM counts). A hybrid set of FEC data was also considered, which involved counts obtained using the SCFT with a 1 epg sensitivity, as well as the other 15EPG_McM counts that were greater than or equal to the thresholds mentioned in Section 2.2. (hereby referred to as 15EPG_McM_SCFT data). As a result, there were four possible sets of data produced for each individual treatment group, for each farm involved. For both Day 0 and Day 14 data, a total of 304 data sets were considered for analysis (i.e. 76 data sets were considered for each diagnostic sensitivity grouping).

In accordance, with the recommended WAAVP guidelines, Coles et al., 1992, Coles et al., 2006, any cattle for which FECs were not obtained either on Day 0, or Day 14, were removed from the final data set and were not included in the analysis. Any cattle that were mis-dosed (as recorded by the veterinarian at the time of treatment), e.g. anthelmintic was rejected by animals after administration or animal movement caused only a partial dose of an anthelmintic being received, which under both circumstances animals received a re-dosing with a full dose as per standard veterinary practice, were also excluded from this analysis. Where duplicate samples were obtained through the experimental process, the first set of FECs recorded was used as part of the analysis in order to ensure independence between the egg counts obtained per animal. Overall, 2501 cattle were sampled during the FECRTs over 52 farms, and of these, 2175 animals results were used in the analysis.

All analyses were carried out using *RStudio* software (version 0.98.994 along with R software version 3.1.1.) and statistical tests which feature as part of this study were carried out at a 5% significance level.

### 2.4 Statistical analyses

#### 2.4.1 Faecal Egg Count Reduction Test (FECRT)

Methods for calculating the FECRT involve determining the arithmetic group mean FEC on Day 0, and/or Day 14, and calculating the percentage reduction and the 95% confidence intervals for treated and untreated groups of animals. Using the World Association for the Advancement of Veterinary Parasitology (WAAVP) method as described by Coles et al. (2006), which is based on faecal samples collected on Day 14, post-treatment, from a treated and an untreated control group (Pepper et al., 2003, McKenna, 2006, Barrerea et al., 2013, Falzon et al., 2013), the following percentage reduction is considered:

(1) $$100\left(1-\;\frac{T_{14}}{C_{14}}\right)\%$$

where $T_{14}$ and $C_{14}$ are the arithmetic group sample mean FECs collected on Day 14, after treatment administration, from the treatment and control groups respectively.

The corresponding 100(1−∝)% confidence interval, where 0<∝ <1, derived using the Delta method (Hosmer et al., 2008, Greene, 2012), which provides large sample approximations for the variance of the *ln*-transformed ratio of means (where *ln* denotes the natural logarithm) and the relevant data are assumed to be normal, for the percentage reduction estimate eq(1) is:

(2) $$100\left(1-\frac{T_{14}}{C_{14}}\exp\left(\pm t_{\left(n_{t}+n_{c}-2\right)}\sqrt{\frac{s_{t}^{2}}{n_{t}T_{14}^{2}}+\frac{s_{c}^{2}}{n_{c}C_{14}^{2}}}\right)\right)\%$$

where, $s_{t}^{2}$, $n_{t}$, $s_{c}^{2}$ and $n_{c}$ represent the sample variances and group sizes for the treated and control groups at Day 14, respectively and where $t_{\left(n_{t}+n_{c}-2\right)}$ is the $\frac{\propto }{2}$ upper-tail probability for a Student's t-distribution with $n_{t}+n_{c}-2$ degrees of freedom. According to Coles et al. (1992), anthelmintic resistance is confirmed if the percentage reduction value eq(1) is less than 95% and the lower confidence limit of the confidence interval eq(2) is less than 90%. If only one of these criteria is met; anthelmintic resistance is suspected. It is worth noting that another experimental design has been adopted for conducting a FECRT, i.e. one which only deals with the arithmetic group means of pre- and post-treatment counts from a positive treatment group (denoted as $T_{0}$ and $T_{14}$ respectively) and has been widely adopted due to the convenience of not having to include a control group (Kochapakdee et al., 1995, Lyndal-Murphy et al., 2010, Levecke et al., 2012, Vidyashankar et al., 2012, Lester et al., 2013, Geurden et al., 2015), for which the following percentage reduction is considered in calculations:

(3) $$100\left(1-\;\frac{T_{14}}{T_{0}}\right)\%$$

The corresponding 100(1−∝)% confidence interval for the percentage reduction eq(3), which has been mathematically derived as part of this study, is then:

(4) $$100\left(1-\frac{T_{14}}{T_{0}}\exp\left(\pm t_{\left(n_{t}-1\right)}\sqrt{\frac{s_{t_{14}}^{2}}{n_{t}T_{14}^{2}}+\frac{s_{t_{0}}^{2}}{n_{t}T_{0}^{2}}-\;\frac{2\rho \left(\ln\left(t_{0}\right),\ln\left(t_{14}\right)\right)s_{\text{ln}\left(t_{0}\right)}s_{\text{ln}\left(t_{14}\right)}}{n_{t}}}\right)\right)\%$$

where, $s_{t_{14}}^{2}$ and $s_{t_{0}}^{2}$ represent the sample variances for the positive treatment group Day 14 ($t_{14}$) and Day 0 ($t_{0}$) FEC data, respectively, $n_{t}$ represents the positive treatment group sample size, $\rho \left(\ln\left(t_{0}\right),\ln\left(t_{14}\right)\right)$ represents the Pearson's correlation coefficient between the *ln-*transformed positive treatment group Day 0 and 14 FEC data, $s_{\text{ln}\left(t_{0}\right)}$ and $s_{\text{ln}\left(t_{14}\right)}$ are the sample standard deviations of the *ln-*transformed positive treatment group Day 0 and 14 FEC data, respectively and where $t_{\left(n_{t}-1\right)}$ is the $\frac{\propto }{2}$ upper-tail probability for a Student's t-distribution with $n_{t}-1$ degrees of freedom.

Some communications also feature arithmetic group means of pre- and post-treatment counts from a control group (denoted as $C_{0}$ and $C_{14}$ respectively) when carrying out a FECRT (Dash et al., 1988, Torgerson et al., 2005, Taylor et al., 2009, Dobson et al., 2012, Lyndal-Murphy et al., 2014), where the following percentage reduction is considered:

(5) $$100\left(1-\;\frac{T_{14}C_{0}}{T_{0}C_{14}}\right)\%$$

The associated 100(1−∝)% confidence interval for eq(5) is an extension of the confidence interval eq(4), where the standard error is derived to be of a similar form:

$$t_{\left(n_{t}+n_{c}-2\right)}\sqrt{\frac{s_{t_{14}}^{2}}{n_{t}T_{14}^{2}}+\frac{s_{t_{0}}^{2}}{n_{t}T_{0}^{2}}-\;\frac{2\rho \left(\ln\left(t_{0}\right),\ln\left(t_{14}\right)\right)s_{\text{ln}\left(t_{0}\right)}s_{\text{ln}\left(t_{14}\right)}}{n_{t}}+\;\frac{s_{c_{14}}^{2}}{n_{c}C_{14}^{2}}+\frac{s_{c_{0}}^{2}}{n_{c}C_{0}^{2}}-\;\frac{2\rho \left(\ln\left(c_{0}\right),\ln\left(c_{14}\right)\right)s_{\text{ln}\left(c_{0}\right)}s_{\text{ln}\left(c_{14}\right)}}{n_{c}}}$$

where, $s_{c_{14}}^{2}$ and $s_{c_{0}}^{2}$ represent the sample variances for the negative control group Day 14 ($c_{14}$) and Day 0 ($c_{0}$) FEC data, respectively, $n_{c}$ represents the negative control group sample size, $\rho \left(\ln\left(c_{0}\right),\ln\left(c_{14}\right)\right)$ represents the Pearson's correlation coefficient between the *ln-*transformed negative control group Day 0 and 14 FEC data, $s_{\text{ln}\left(c_{0}\right)}$ and $s_{\text{ln}\left(c_{14}\right)}$ are the sample standard deviations of the *ln-*transformed negative control group Day 0 and 14 FEC data.

It is also worth noting that confidence intervals and credible intervals for percentage reduction estimates eq(1), eq(3) and eq(5) can be obtained using Bootstrapping (Efron and Tibshirani, 1993) and Bayesian frameworks (Denwood, 2010, Torgerson et al., 2014) for different livestock species, since generating these types of intervals using such frameworks is not necessarily dependent on assumptions of normality, which the Delta method utilises.

#### 2.4.2 Assessing normality of original and transformed FEC data

An assessment of normality was conducted by using a *Shapiro-Wilk* normality test in which the null hypothesis is that the data follow a normal distribution (Royston, 1982a, Royston, 1982b, Royston, 1995, Razali and Wah, 2011). Each original data set was assessed for normality and the *ln(x* + *1)* (where *x* is defined as a FEC), the square-root and $x^{\frac{2}{3}}$ power transformations were also applied and assessed for normality. These transformations were used in an attempt to correct the usual skewness present in FEC data which are commonly used when dealing with discrete count data, especially when zero counts are present (Zar, 1996). In the case of an inconclusive result being obtained from the normality test, this could be due to either the data set being too small for the test to be conducted or the counts present in the data set were all the same value.

#### 2.4.3 Compound distributions

Compound distributions result from allowing distributions and their associated parameters, such as central tendencies, to follow other distributions (Upton and Cook, 2011). For example, if a discrete random variable Y were to follow a Poisson distribution with central tendency μ and μ ∼ GA(1, $\sigma^{\frac{1}{2}}$), for which GA denotes the gamma distribution, then this gives rise to the negative binomial (i.e. gamma-Poisson) distribution that has parameters mean μ and scale parameter σ. In fact, the negative binomial is often referred to as a gamma-Poisson distribution and is used often in parasitology to describe egg count data where counts are highly aggregated or over-dispersed, i.e. the variation present in the egg count data is greater than expected (Levecke et al., 2012). According to Rigby et al. (2014), certain compound distributions can also account for excess zeros such as *zero inflated* distributions (ZIDs). For these we consider Y, i.e. cattle FECs, that can exhibit a greater proportion of zeros than a certain discrete count distribution, $Y_{1}$ (Zuur et al., 2009, Rigby et al., 2014). The probability mass function is given by:

$$\begin{array}{c} P\left(Y=0\right)=\upsilon +\left(1-\upsilon \right)P\left(Y_{1}=0\right)\\ P\left(Y=y\right)=\left(1-\upsilon \right)P\left(Y_{1}=y\right) \end{array}$$

where 0< υ *<*1 and *y* = 1,2, …(Zuur et al., 2009, Rigby et al., 2014). The parameter υ represents the proportion of zeros present in the data. From this, Bohning et al. (1999) tell us that the maximum likelihood estimator $\mu_{1}$ say, of the ZID is:

(6) $$\mu_{1}=\;\frac{\mu \;}{\left(1-\;\upsilon \right)}$$

The value $\mu_{1}$ can be determined as the arithmetic group mean of FECs divided by the proportion of non-zero counts present in count data. We can also consider *zero adjusted* distributions (ZADs), which allow us to consider a greater or less proportion of zeros than would be obtained from a certain discrete count distribution thus, ZIDs are considered to be a specific case of ZADs.

#### 2.4.4 Compound distributions fitted to FEC data

Thirteen relevant compound distributions were fitted to the FEC data using the General Additive Models for Location, Scale and Shape (GAMLSS) package in *RStudio* (Rigby and Stasinopoulos, 2005, Stasinopoulos and Rigby, 2007). The following distributions were chosen to be fitted since they included a location parameter, i.e. central tendency, that could be estimated: the Poisson, negative binomial (two parameterisations, Type I and II and hence referred to as NBI and NBII, respectively), geometric, Poisson inverse-Gaussian, Sichel (i.e. a generalized inverse-Gaussian Poisson) and Delaporte (i.e. a shifted gamma-Poisson) distributions. All of these distributions had a central tendency μ (arithmetic mean estimate $\bar{x}$) that could be estimated. With respect to ZIDs, two parameterisations of a zero inflated Poisson, one with a central tendency $\mu_{1}$(referred to as ZIPI) and the other μ (referred to as ZIPII), a zero inflated Poisson inverse-Gaussian and a zero inflated negative binomial distribution (both having central tendencies $\mu_{1}$) were fitted. A zero adjusted Poisson and a zero adjusted NBI distribution were also fitted. Fitting was undertaken using the *gamlssML()* function, which estimated a distribution's relevant parameters by maximum likelihood (Wimmer and Altmann, 1999, Johnson et al., 2005, Rigby et al., 2014).

The fit of each distribution was assessed using *Akaike's Information Criterion* (AIC) (Akaike, 1973, Zuur et al., 2009); which was evaluated for each distribution fitted, and whereby the lowest AIC value obtained indicated the distribution of best representation. The AIC is defined as

AIC=−2(log-likelihood)+2(NumberofParametersfeaturedindistribution).

**Fig. 1:**
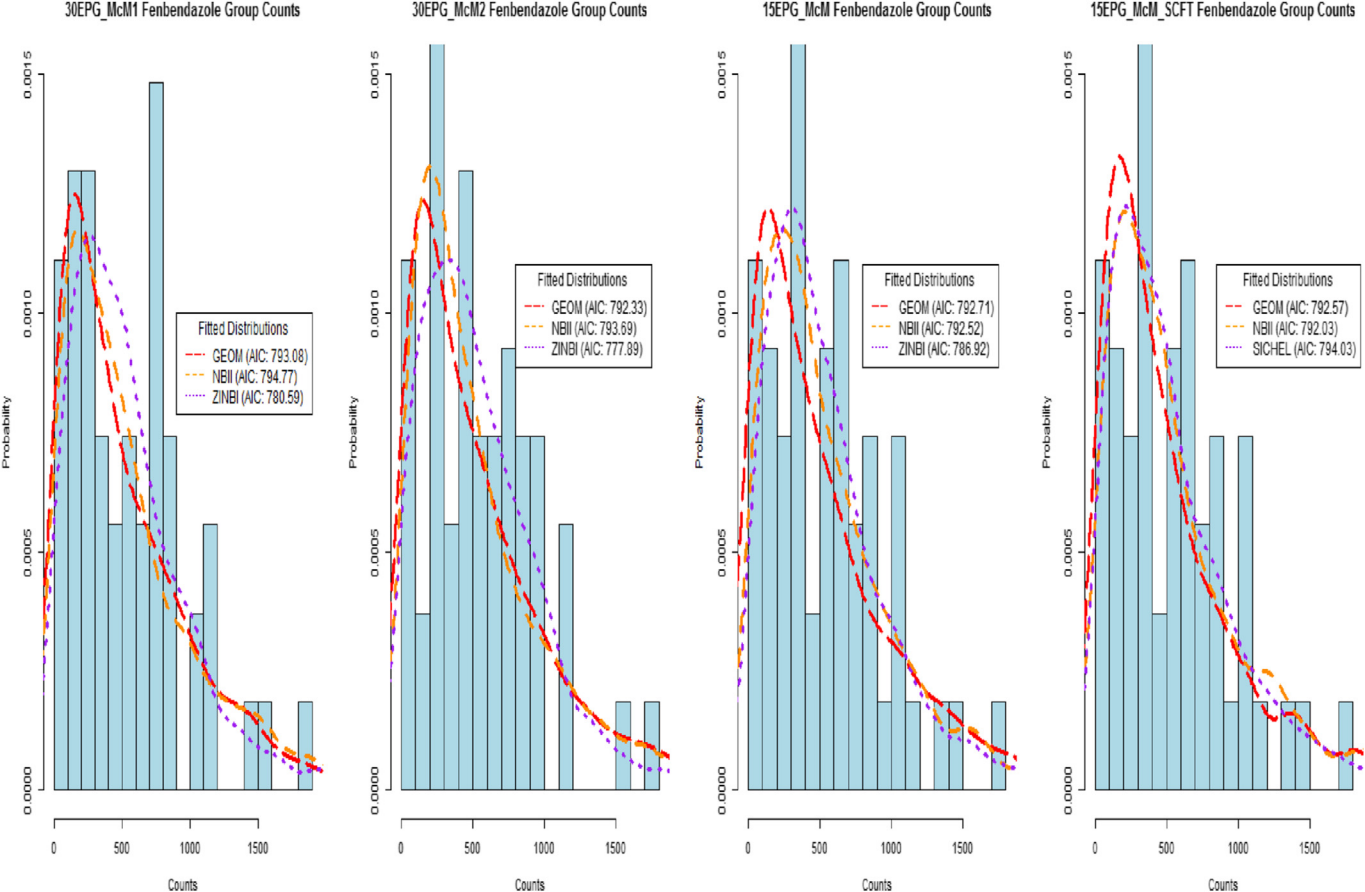
Farm E32 fenbendazole Day 0 FEC data with example fitted distributions and their associated AIC values.

**Fig. 2:**
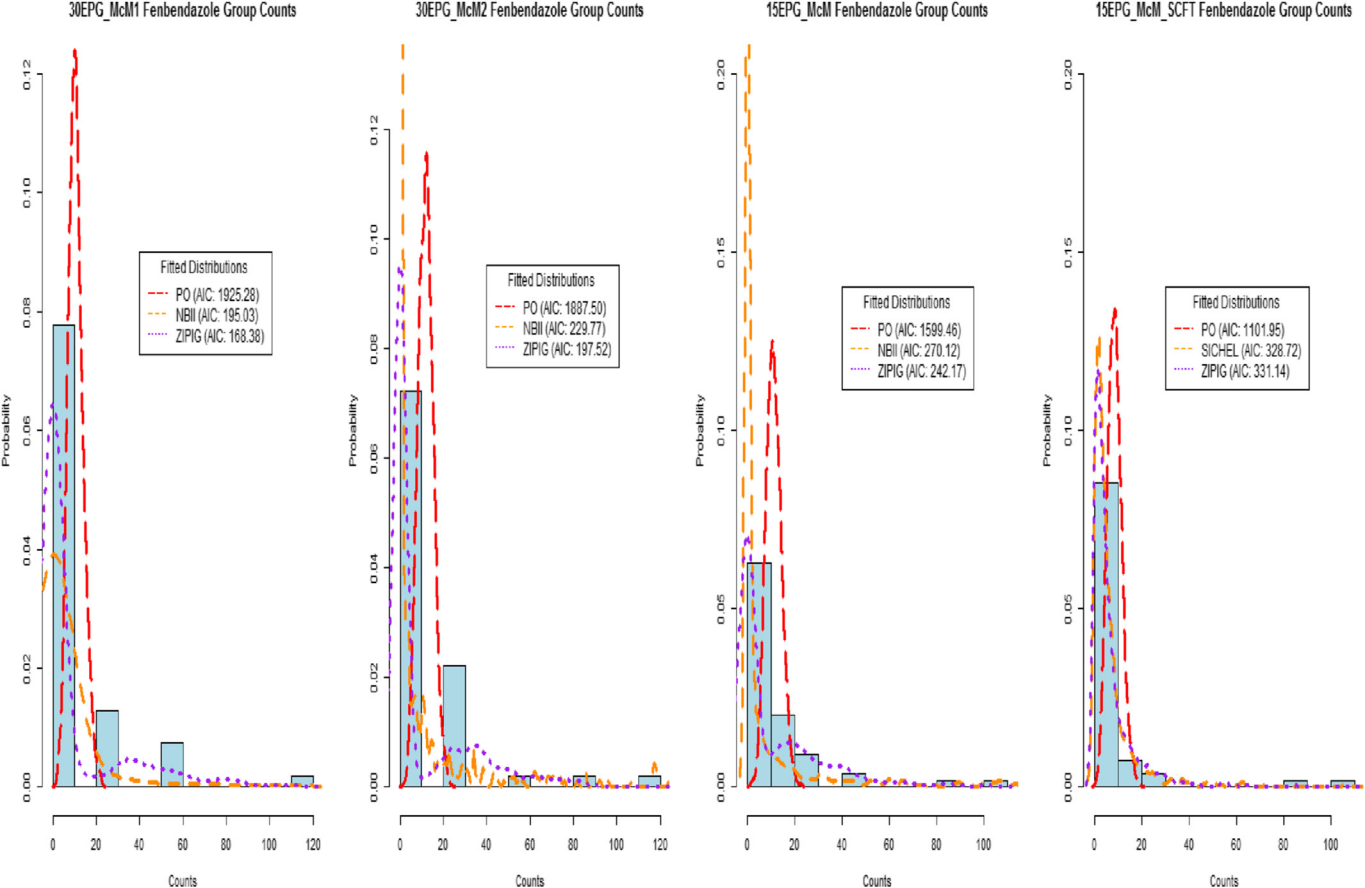
Farm E32 fenbendazole Day 14 FEC data with example fitted distributions and their associated AIC values.

The AIC value takes into account the fit of the distribution to the data (i.e. the *log-likelihood)* whilst at the same time penalising the fit of the distribution by adding twice the number of parameters (Rigby et al., 2014). However, if no distribution could be fitted to a particular data set (i.e. due to the counts being all of the same value of zero), the fit was recorded and classed as inconclusive and this was also summarised where appropriate. As a means of observing the goodness of fit for the selection of distributions considered, the fenbendazole Day 0 and Day 14 treatment group FEC data from an example farm (farm E32) are displayed in Fig. 1, Fig. 2. Examples of fitted distributions based on 1000 simulated random samples are also included in these figures, where these samples were simulated from distributions using parameters estimated from the *gamlssML()* function. Their associated AIC values are displayed - where the lowest valued AIC displayed in these figures indicates the distribution of best fit for that particular set of data.

#### 2.4.5 Percentage reduction and confidence limit comparisons

Comparisons of the percentage reductions eq(1), eq(3) and eq(5) and their associated 95% upper confidence limits (UCLs) and lower confidence limits (LCLs) were made using estimated arithmetic group means, $\bar{x}$, and the central tendency estimates from the appropriate best-fitted distributions across all four types of FEC data. Depending on the results of the assessment of normality as described in Section 2.4.2., if the majority of the original Day 0 and Day 14 FEC data were concluded as being normally distributed, then confidence intervals were estimated using the Delta method, otherwise 95% bootstrapped percentile intervals were estimated from 5000 iterations for each of the Day 0 and Day 14 control and positive treatment group data, where every combination of the 5000 estimates obtained for each set of data was considered, resulting in a sampling distribution of 2.5×$10^{7}$ percentage reduction estimates, from which the percentile intervals were derived from. When considering distributions that were classed as inconclusive, the central tendency μ was used to represent the counts involved that all had the same value of zero, as this is the only appropriate central tendency that can be used to represent these data.

## 3 Results

### 3.1 Assessing normality of FEC data

Table 1 highlights the normality results of the original 304 Day 0 and Day 14 FEC data and the transformed versions of each data set involved. 87.5% and 93.8% of the original Day 0 and Day 14 FEC data were, respectively, classed as non-normal. With respect to the transformed versions of these data, apart from the square-root transformed Day 0 data, the majority of the transformed versions of the Day 0 and Day 14 FEC data were also considered to be non-normal.

### 3.2 Fitted distributions

A total of 304 sets of FEC data were obtained from Day 0 and a summary of the frequencies of the best-fitting distributions for each of the diagnostic sensitivity groupings are shown in Table 2. With respect to the Day 14 data, a similar summary of the frequencies is displayed in Table 3. From both tables we observe a high occurrence ZIDs with central tendency eq(6), being reported as the best-fitting types of distributions in the majority of the diagnostic sensitivity groupings, except for the 15EPG_McM_SCFT data; the most common best-fitted distributions were those associated with the negative binomial distribution, with central tendency μ. Fig. 1, Fig. 2 display the fenbendazole Day 0 and Day 14 treatment group FEC data from farm E32, respectively, and examples of fitted distributions (based on simulated data from estimated parameters) along with their associated AIC values. From these figures, we can observe that zero inflated distributions represent the data obtained using 30 or 15 epg sensitivities very well, particularly for Day 14 FEC data, and how well distributions associated with the negative binomial fit the data in comparison to one another for the 15EPG_McM_SCFT data.

### 3.3 Percentage reduction and confidence limit comparisons

**Fig. 3:**
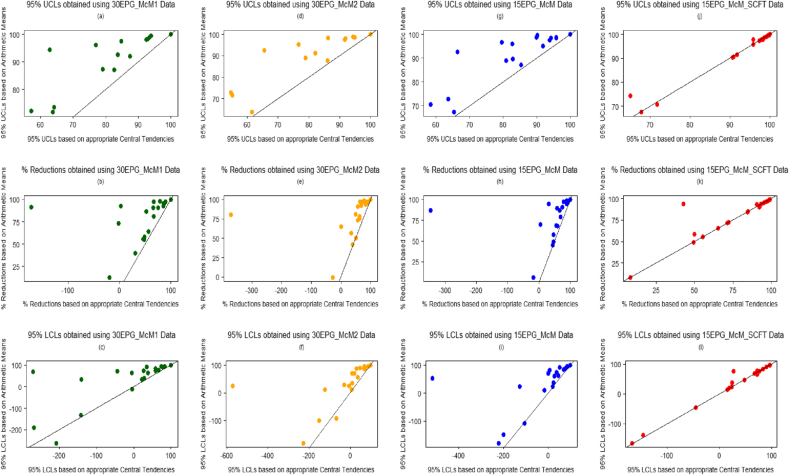
Comparison of $100\left(1-\;\frac{T_{14}}{C_{14}}\right)$% estimates and corresponding 95% UCLs and LCLs obtained using FEC data (central tendency estimates from best-fitted distributions used vs. arithmetic group means used). (a)–(c) based on 30EPG_McM1 data, (d)-(f) based on 30EPG_McM2 data, (g)-(i) based on 15EPG_McM data and (j)-(l) based on 15EPG_McM_SCFT data.

**Fig. 4:**
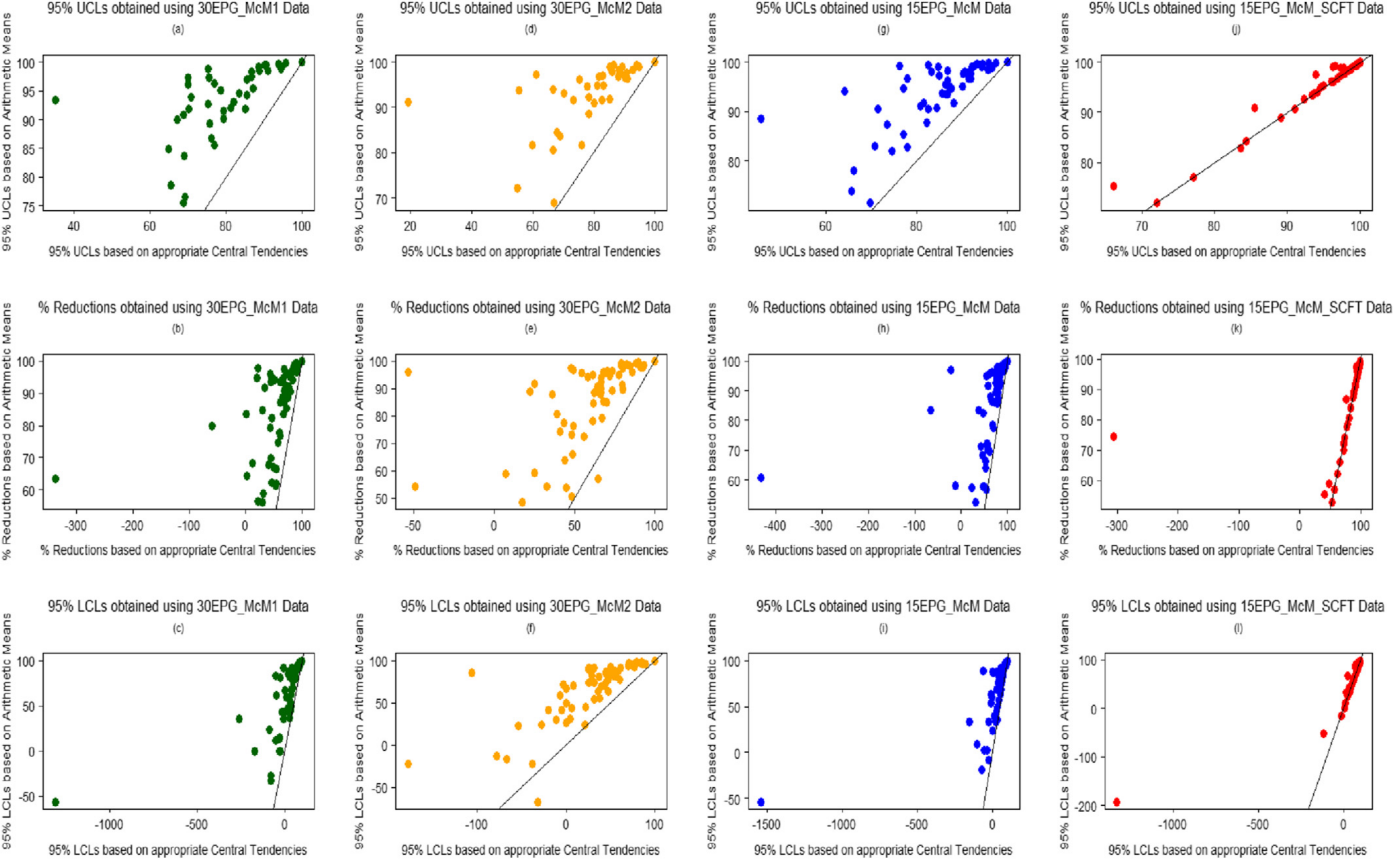
Comparison of $100\left(1-\;\frac{T_{14}}{T_{0}}\right)$% estimates and corresponding 95% UCLs and LCLs obtained using FEC data (central tendency estimates from best-fitted distributions used vs. arithmetic group means used). (a)–(c) based on 30EPG_McM1 data, (d)-(f) based on 30EPG_McM2 data, (g)-(i) based on 15EPG_McM data and (j)-(l) based on 15EPG_McM_SCFT data.

**Fig. 5:**
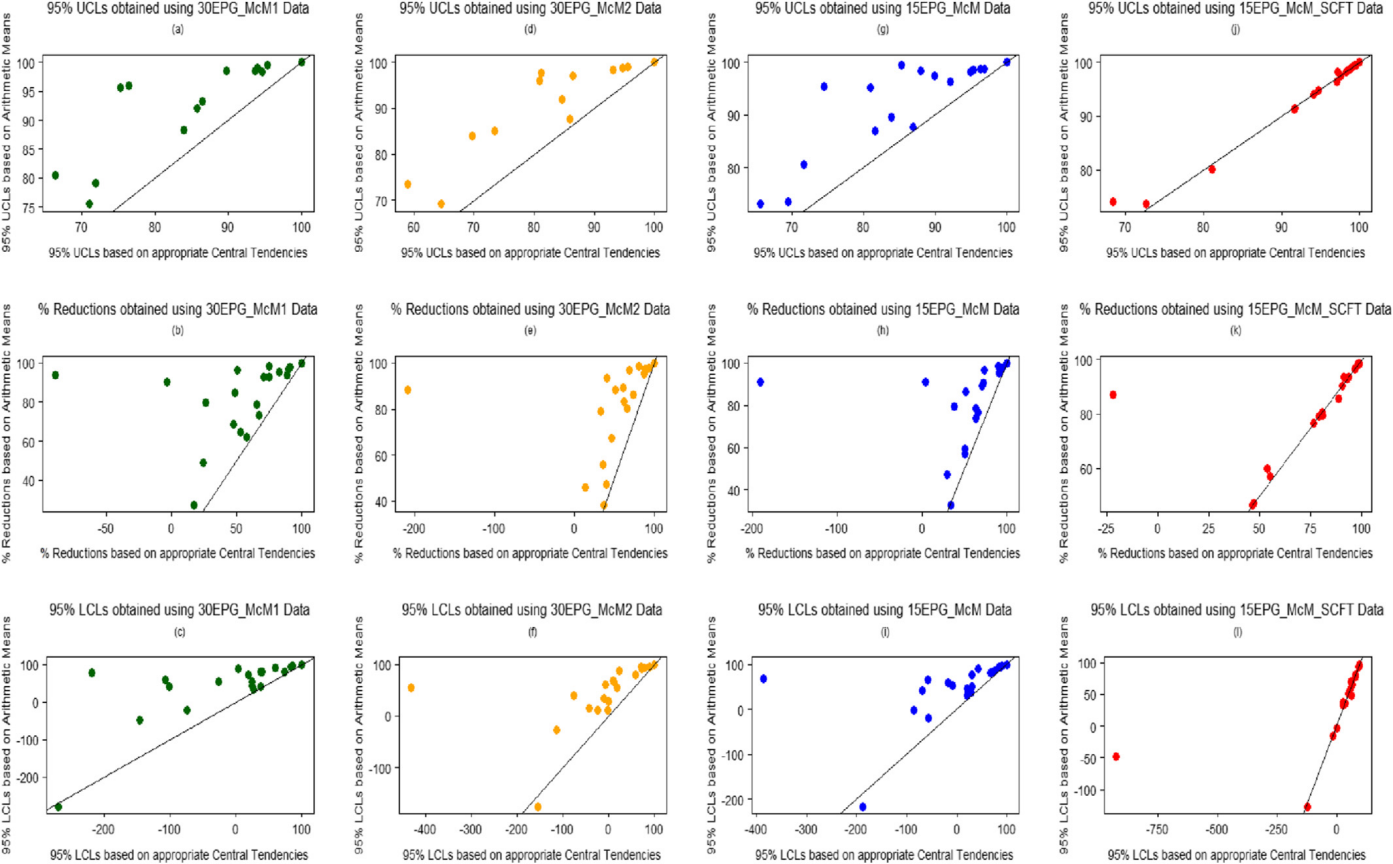
Comparison of $100\left(1-\;\frac{C_{0}T_{14}}{C_{14}T_{0}}\right)$% estimates and corresponding 95% UCLs and LCLs obtained using FEC data (central tendency estimates from best-fitted distributions used vs. arithmetic group means used). (a)–(c) based on 30EPG_McM1 data, (d)-(f) based on 30EPG_McM2 data, (g)-(i) based on 15EPG_McM data and (j)-(l) based on 15EPG_McM_SCFT data.

Percentage reduction estimates eq(1), eq(3) and eq(5) and their associated 95% Bootstrapped percentile UCLs and LCLs were evaluated and compared using the arithmetic group mean $\bar{x}$ of the data, and using the central tendencies of the best-fitted distributions. Fig. 3, Fig. 5 give visual representations of the comparison of the available 20 eq(1), and eq(5) percentage reductions and their associated confidence limits, respectively, using FEC data from the four diagnostic sensitivities. Fig. 4 displays the comparison of the 61 eq(3) percentage reductions and 95% confidence limits. The straight lines featuring in these plots represent the scenario of either the percentage reductions (figures labelled (b), (e), (h) and (k)), UCLs (figures labelled (a), (d), (g) and (j)) or LCLs (figures labelled (c), (f), (i) and (l)) being equal when evaluated using both sets of estimates.

Overall, for each type of FECRT calculation method, the percentage reduction and the associated confidence limits estimated for FEC data obtained using 30 or 15 epg sensitivities; using arithmetic means resulted in higher valued percentage reductions and interval estimates being obtained, in comparison to those estimated using the central tendencies of the best-fitting distributions, i.e. zero inflated distributions. This was also the case for the comparisons that could be considered as outliers in Fig. 3, Fig. 4, Fig. 5, since these points lie above the straight lines in these figures. However, there was good agreement between the percentage reductions and confidence limits estimated using arithmetic means and central tendencies of the best-fitting distributions when considering the 15EPG_McM_SCFT data; since the majority of comparisons lie on the straight lines in the associated figures.

## 4 Discussion

The FECRT remains a widely used field test for anthelminthic efficacy, despite it never having been validated against slaughter studies. This study provides insight into the potential statistical distributions that could be applied to cattle FEC data to reduce over-interpretation of FECRT data.

The original 304 Day 0 and Day 14 FEC data sets and transformed versions of these were assessed for normality, since confidence intervals currently recommended to be used in a FECRT are derived assuming relevant data to be normal to obtain approximate estimates for the *ln*-transformed ratio of means of FEC data and its associated variance. For smaller sample sizes (<30), the Student's t-distribution is utilised to generate confidence intervals since it provides a more conservative estimate (i.e. confidence limits are wider) in comparison with the standard normal distribution. Furthermore, the transformations were used in an attempt to correct the usual skewness present in FEC data, to obtain data that would be considered as symmetric (Zar, 1996, Torgerson et al., 2005, Vidyashankar et al., 2007).

The majority of the original and transformed data sets, both on Day 0 and Day 14, were found to be non-normal via the Shapiro-Wilk normality test. As a result, it would not be recommended to use confidence intervals that are based on large sample approximations that assume normality of relevant data. Moreover, some of the confidence intervals derived by the Delta Method, rely on correlations of natural logarithmic-transformed FEC data being evaluated; but this would not be possible if zero-valued FECs were obtained as part of anthelmintic studies. In fact, given the nature of these data; confidence or credible intervals would be more suitably estimated using alternative methods such as Bootstrapping or a Bayesian approach since generating these types of intervals from such frameworks is not necessarily dependent on assumptions of normality. Bootstrapping is a computer intensive and data driven technique that involves re-sampling observed data (Efron and Tibshirani, 1993) and is generally regarded by the veterinary and parasitological communities to potentially offer a simple, accessible and robust method to generate and infer confidence intervals for percentage reduction estimates, even in the presence of small sample sizes (Cabaret and Berrag, 2004, Lester and Matthews, 2013, Lester et al., 2013). Bayesian Statistics lead us to work with a distribution for the parameters of interest (as opposed to fixing parameters to be estimated from data) for which credible intervals can be generated, and is the basis for subsequent inference within the Bayesian paradigm (Rice, 2007). A Bayesian approach to analysing data offers benefits such as the usual normality assumption within statistical models being removed and unrealistic assumptions and simplifications being avoided when considering data. Research into the application of Bayesian methods when investigating apparent anthelmintic efficacy and resistance has been conducted, but mainly using equine faecal egg count data (Denwood, 2010, Denwood et al., 2010). However, Bayesian approaches are being employed in more recent sheep and cattle studies (Denwood et al., 2008, Dobson et al., 2012, Busin et al., 2013, Geurden et al., 2015). In fact, Denwood (2010) and Torgerson et al. (2014) suggest the use of Bayesian inference when dealing with FEC data; but Matthews (2014) highlights that a limitation to adopting Bayesian methods in analysing FEC data is the ability to use advanced statistical programmes, which the layperson may not be familiar with. The work presented here makes use of maximum likelihood estimation through the GAMLSS package, which is able to estimate distributional parameters, other than central tendencies such as variability, proportion of zeros etc., without the extra computational intensity that Bayesian inference can involve.

The results overall suggest that by using real cattle FECs obtained by sensitive counting techniques (such as the SCFT with a diagnostic sensitivity of 1 epg), distributions associated with and including the negative binomial distribution could be recommended to represent these types of data. Hence, percentage reductions and confidence limits could be estimated using arithmetic group means (i.e. the central tendency estimates associated with these distributions) in order to evaluate apparent anthelmintic efficacy. If cattle FEC data are obtained with less sensitive counting techniques (such as the McMaster technique with diagnostic sensitivities of 30 epg or 15 epg), ZIDs are recommended to represent these data, with central tendency $\mu_{1}$ being used when calculating percentage reductions and confidence limits, due to excess zeros being produced by the counting techniques employed. As a result, this study demonstrates that the diagnostic sensitivities used in egg counting techniques influence the distribution of best representation for FEC data. For cattle, this is a consistent result with the study of El-Abdellati et al. (2010), who also reported that detection limits of counting techniques used in experimental studies are confounding factors of major importance when investigating anthelmintic resistance.

With respect to ZIDs, these distributions (more specifically zero inflated negative binomial distributions) have been used in worm egg count simulation studies involving sheep (Denwood et al., 2008) and horses (Denwood, 2010, Denwood et al., 2010). The present study compliments these earlier studies and advocates the use of ZIDs for representing real cattle FECs obtained using less sensitive counting techniques, but with alternative estimators being utilised. One could argue though, that in the present study there were animals involved whose FECs were less than the 100 epg threshold recommended by Coles et al. (2006) and so this introduces the need for ZIDs. However, the level of egg excretion is generally low and highly aggregated in cattle, i.e. the majority of cattle will be shedding low numbers of eggs in their faeces as well as few animals shedding a higher number of eggs (Demeler et al., 2009, El-Abdellati et al., 2010, Levecke et al., 2012) and is the reason as to why cattle with FECs less than 100 epg were included in this study.

The choice of which central tendencies should be used to represent FEC data, and therefore be used as part of a FECRT, has been long debated in veterinary parasitology research, despite the fact that the choice of central tendency depends on the distributions of best fit. For instance, the use of geometric means has been suggested previously (Presidente, 1985, Mejia et al., 2003), but the use of arithmetic means has been more widely adopted (Dash et al., 1988, Dobson et al., 2009) in anthelmintic efficacy studies. In fact, Geurden et al. (2015) investigated anthelmintic efficacy in cattle in Europe, where egg counts were obtained using a diagnostic sensitivity of 12.5 epg (and 15 epg in one country) and arithmetic means were used to calculate percentage reductions. Our study, however, recommends the use of the central tendency $\mu_{1}$ for FECRT calculations as opposed to the use of arithmetic means when dealing with ZIDs, on the basis that this is the maximum likelihood estimator for these types of distributions. It is worth noting that this estimator is greater than the value of μ alone and can take account of the non-zero counts and higher-valued data points present in zero inflated data (in fact it is often the case with FEC data that a small number of individual animals will be shedding high numbers of helminth eggs in their faeces), which may not be accounted for when locations such as the arithmetic mean are used (due to zero inflation of potential false zeros decreasing the value of μ to a location closer to the value of zero).

However, with this recommendation in mind, it naturally leads us to ask for which diagnostic sensitivities between 15 epg and 1 epg do we start accepting distributions, such as the negative binomial, being the better representation in comparison to ZIDs? As part of the current study we are unable to answer this question, but this could be investigated as part of future studies.

Percentage reductions and their associated 95% UCLs and LCLs were evaluated using arithmetic group means and using the central tendency estimates of the best-fitted distributions for each of the different types of FEC data. With regards to FEC data obtained using 30 or 15 epg sensitivities; using the central tendency estimates of the best-fitted distributions - for which the central tendency $\mu_{1}$ was often utilized since the majority of these distributions were ZIDs - resulted in lower percentage reductions and confidence limits being obtained, in comparison to using arithmetic means. As a result, for FEC data obtained by less sensitive counting techniques, an anthelmintic could be interpreted as over-performing (bearing in mind that a FECRT gives an indirect indication of efficacy) when arithmetic group mean estimates are used in the presence of zero inflated data. Based on the hybrid sets of data, there was good agreement between the percentage reductions and confidence limits estimated using both arithmetic means and central tendencies of the best-fitting distributions due to the fact that the majority of these data were best represented by distributions whose central tendency was the arithmetic mean, i.e. those associated with the negative binomial distribution.

## 5 Conclusion

The FECRT remains a widely used, but unvalidated field test for anthelminthic efficacy, and this study does not change this fact. That said, the results of this study have given insight into the potential discrete count distributions that best represent cattle FEC data and which parameters and types of intervals that could be used when performing a FECRT that can reduce the risk of misinterpreting/misclassifying the apparent efficacy status of farms. The results obtained here support the use of the $\mu_{1}$ estimate when calculating percentage reductions, instead of using arithmetic means for FECs obtained by less sensitive counting techniques (i.e. McMaster 30 or 15 epg); otherwise, an anthelmintic could be thought of as over-performing when arithmetic mean estimates are used in the presence of zero-inflated data. When sensitive counting techniques are used (i.e. with a 1 epg sensitivity) to obtain FEC data, percentage reductions are recommended to be evaluated using arithmetic group mean estimates. However, in the case of an anthelmintic appearing to be fully effective and reducing all FECs to zero counts post-treatment, in spite of the sensitivity of the counting technique used; the only appropriate central tendency for this set of data is the arithmetic mean (effectively the value of zero). Further, it would not be recommended to use 95% confidence intervals based on large sample, normal approximations for percentage reduction estimates since majority of FEC data are considered to be of a non-normal nature, even upon transformation. It is therefore recommended that relevant intervals for percentage reduction estimates be obtained using a Bootstrap or Bayesian framework, in order to obtain reliable estimates and interpretations of apparent anthelmintic efficacy in studies, for the foreseeable future.

## Financial support

This work was supported by funding from the Engineering and Physical Sciences Research Council (PhD Scholarship to J.L., grant numbers EP/L505080/1 and EP/K503174/1) and by the Veterinary Medicines Directorate (VM0522 Project).

## Figures and Tables

**Table 1:** *Shapiro-Wilk* Normality test results for Day 0 and Day 14 data and the various transformations applied to these data.

	Original Data		ln(x+1) Data		Square-Root Transformed Data		$x^{\frac{2}{3}}$ Transformed Data	
	Day 0	Day 14	Day 0	Day 14	Day 0	Day 14	Day 0	Day 14
Data sets that were considered normal	38 (12.50%)	12 (3.90%)	78 (25.70%)	24 (7.90%)	154 (50.70%)	41 (13.50%)	104 (34.20%)	29 (9.50%)
Data sets that were considered non-normal	266 (87.50%)	285 (93.80%)	226 (74.30%)	273 (89.80%)	150 (49.30%)	256 (84.20%)	200 (65.80%)	268 (88.20%)
Data sets that were inconclusive	0 (0%)	7 (2.30%)	0 (0%)	7 (2.30%)	0 (0%)	7 (2.30%)	0 (0%)	7 (2.30%)

**Table 2:** Frequencies (and relative frequencies) of the best-fitting distributions for Day 0 data sets, categorised by the four diagnostic sensitivity groups.

Best Fitting Distributions^a^	30EPG_MCM1 Data (%)	30EPG_MCM2 Data (%)	15EPG_McM Data (%)	15EPG_McM_SCFT Data (%)
DEL	0 (0.00%)	0 (0.00%)	0 (0.00%)	6 (7.89%)
GEOM	2 (2.63%)	4 (5.26%)	8 (10.53%)	21 (27.63%)
NBII	4 (5.26%)	3 (3.95%)	4 (5.26%)	21 (27.63%)
PIG	4 (5.26%)	5 (6.58%)	11 (14.47%)	16 (21.05%)
SICHEL	0 (0.00%)	0 (0.00%)	0 (0.00%)	5 (6.58%)
ZINBI	20 (26.32%)	11 (14.47%)	19 (25.00%)	5 (6.58%)
ZIPIG	46 (60.53%)	53 (69.74%)	34 (44.74%)	2 (2.63%)

a DEL = Delaporte, GEOM = Geometric, NBII=Negative Binomial (Type II), PIG=Poisson Inverse-Gaussian, SICHEL=Sichel, ZINBI = Zero Inflated Negative Binomial (Type I), ZIPIG = Zero Inflated Poisson Inverse-Gaussian.

**Table 3:** Frequencies (and relative frequencies) of the best-fitting distributions for Day 14 data sets, categorised by the four diagnostic sensitivity groups.

Best Fitted Distributions^a^	30EPG_MCM1 Data (%)	30EPG_MCM2 Data (%)	15EPG_McM Data (%)	15EPG_McM_SCFT Data (%)
DEL	0 (0.00%)	0 (0.00%)	0 (0.00%)	8 (10.53%)
GEOM	0 (0.00%)	0 (0.00%)	0 (0.00%)	12 (15.79%)
INCONCLUSIVE^b^	3 (3.95%)	2 (2.63%)	2 (2.63%)	0 (0.00%)
NBII	1 (1.32%)	2 (2.63%)	2 (2.63%)	23 (30.26%)
PIG	0 (0.00%)	0 (0.00%)	0 (0.00%)	17 (22.37%)
PO	0 (0.00%)	0 (0.00%)	0 (0.00%)	1 (1.32%)
SICHEL	0 (0.00%)	0 (0.00%)	0 (0.00%)	1 (1.32%)
ZINBI	6 (7.89%)	5 (6.58%)	8 (10.53%)	4 (5.26%)
ZIPI	14 (18.42%)	20 (26.32%)	15 (19.74%)	4 (5.26%)
ZIPIG	52 (68.42%)	47 (61.84%)	49 (64.47%)	6 (7.89%)

a DEL = Delaporte, GEOM = Geometric, NBII=Negative Binomial (Type II), Poisson Inverse-Gaussian, PO=Poisson, SICHEL=Sichel, ZINBI = Zero Inflated Negative Binomial (Type I), ZIPI = Zero Inflated Poisson and ZIPIG = Zero Inflated Poisson Inverse-Gaussian.

b INCONCLUSIVE status refers to all counts being zero.
